# Hypoxia-inducible factor 2α: at the interface between oxygen sensing systems in physiology and pathology

**DOI:** 10.1152/physiol.00043.2024

**Published:** 2025-02-13

**Authors:** Tammie Bishop, Peter J. Ratcliffe

**Affiliations:** Ludwig Cancer Research Department of Physiology, Anatomy and Genetics NDM Research Building Sherrington Building, Old Road Campus Sherrington Road, Roosevelt Drive Oxford, OX1 3PT Oxford, OX3 7FZ; Ludwig Cancer Research Francis Crick Institute, NDM Research Building 1 Midland Road, Old Road Campus London, NW1 1AT Roosevelt Drive, Oxford, OX3 7FZ

## Abstract

More than 100 years after the original descriptions of altitude adaptation, it is now clear that many of these responses are mediated by a specific isoform of the transcription factor hypoxia-inducible factor (HIF-2α). Here, we review this work, including connectivity with the oxygen chemosensitive response itself, and with paraganglioma, a tumour often affecting chemosensitive tissues.

## Introduction and historical notes

In the mid-nineteenth century, Claude Bernard recognized the control of oxygen as part of his founding principle that the stability of the internal environment (*milieu interieur*) was ‘necessary for free and independent life’. However, it was his protégé, Paul Bert, who made the first significant experimental observations on altitude and hypoxia. Bert showed that many of the symptoms that develop at high altitude can be compensated by breathing supplemental oxygen and deduced that they arise from hypoxia rather than the reduced barometric pressure itself (1). It subsequently became clear that levels of oxygen and carbon dioxide in the blood are both capable of controlling breathing. Rather surprisingly, however, in their classical analyses of this, Haldane and Priestley demonstrated that, under normal conditions, control of breathing is almost entirely dependent on pCO_2_ (2). Amongst other observations, they found that pCO_2_ was maintained at a near-constant level irrespective of modest changes in altitude or metabolic stresses such as moderate exercise. Furthermore, even small increases in the arterial pCO_2_ achieved by additions to the inspired atmosphere produced very large increases in ventilation. Nevertheless, consistent with Bert’s observations, Haldane and colleagues did observe that this principle broke down in severe hypoxia, which was associated with increased respiration sufficient to reduce arterial pCO_2_ (2, 3). Haldane and colleagues also noted that this elevation in breathing could be corrected by addition of oxygen to a hypobaric chamber, thus again implicating additional respiratory control by oxygen. Interestingly, however, both measurements in the chamber and measurements over a more protracted period at high altitude (for instance during the Oxford-Yale expedition to Pike’s Peak (14,115ft), Colorado)(4) revealed that this reduction in pCO_2_ increased gradually with the duration of stay at altitude. Puzzlingly, it was also observed that the arterial pCO_2_ returned only gradually to normal on return to sea-level. These observations led Haldane and others to propose that the stimulus was not oxygen *per se*, but the accumulation and dissipation of acid, likely lactic acid, as a consequence of anaerobic cellular respiration. However, no explanatory acidosis was found, neither then nor subsequently.

**Figure 1:**
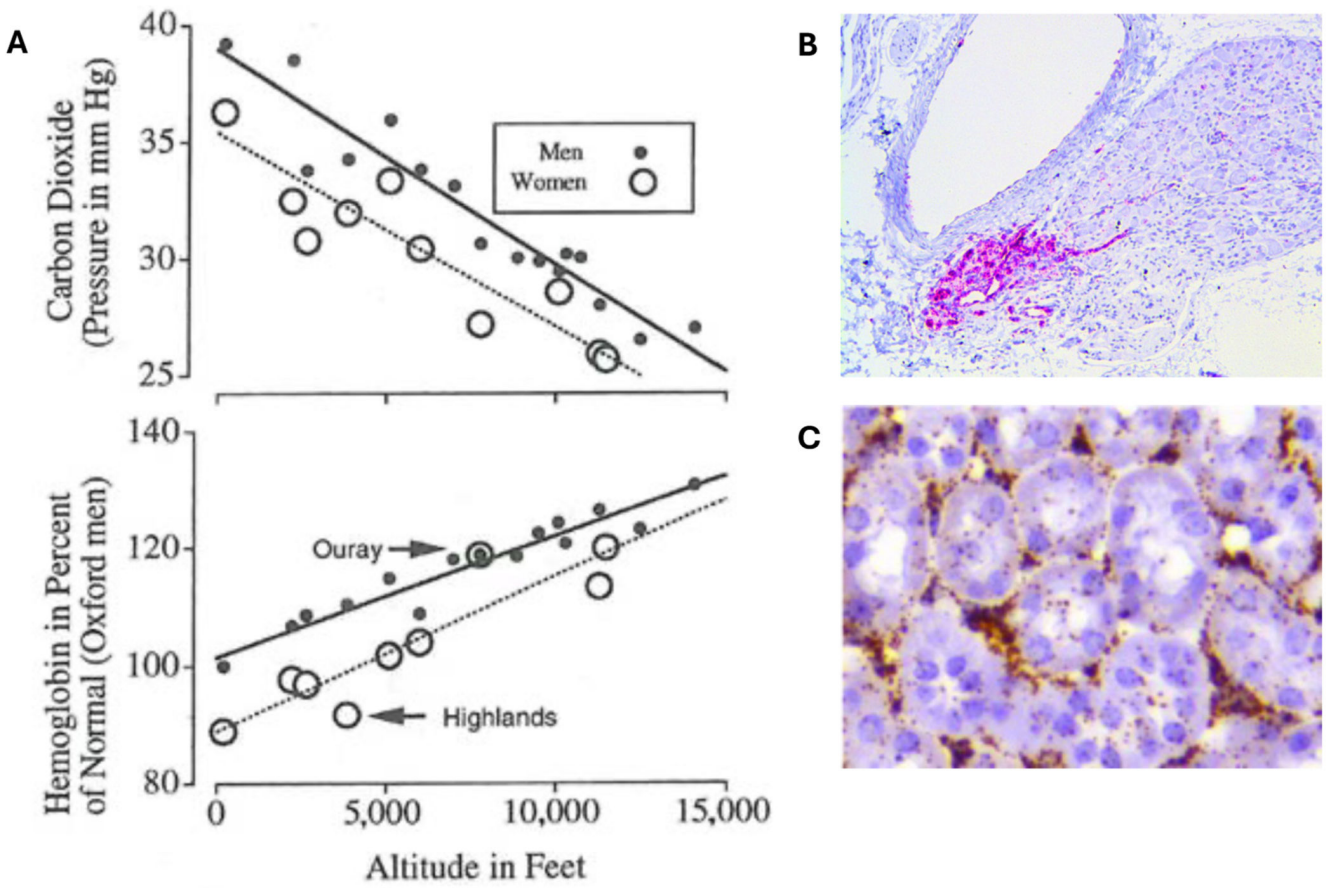
HIF-2α and acclimatisation to altitude. **(A)** Ventilatory acclimatisation (top graph) and erythropoiesis (bottom graph) in populations acclimatised to living in mining towns at altitude. Ouray, Colorado, USA; Highlands, Appalachian Mountains, Pennsylvania, USA. Graphs adapted from FitzGerald, *Philos Trans R Soc Lond B Biol Sci*, 1913. *HIF-2**α*** is necessary for both these adaptations to altitude and is highly expressed at the mRNA level in tissues that mediate these processes: Type I cells of the carotid body **(B)**(*HIF-2**α*** mRNA in pink) and the erythropoietin (EPO) producing renal interstitial fibroblasts **(C)**(*HIF-2**α*** mRNA in brown) of mice. Data from Fielding *et al*., *JPhysiol*, 2018 and unpublished observations.

What Haldane and colleagues observed comprises part of the process of altitude acclimatisation, a phenomenon that is now recognized to be largely a response to hypoxia itself (5). In retrospect, this might have been deduced from the beautifully precise measurements of resting arterial pCO_2_ and blood haemoglobin level made by the female member of Haldane’s party, Mabel FitzGerald. FitzGerald’s measurements were made not at the summit of Pike’s Peak but in the surrounding mining towns at intermediate altitudes, in genetic lowlanders who were fully acclimatised to the altitude at which the measurements were made (6). Her work revealed similarly sensitive monotonic relationships between altitude and increases in blood haemoglobin (with its direct link to oxygen physiology) and between altitude and increases in respiratory sensitivity (as manifest by a reduction in resting arterial pCO_2_)(Figure 1A).

Here we will review some of the evidence that both of these responses (increases in blood haemoglobin and increases in respiratory sensitivity in acclimatised subjects) arise from activation of the hypoxia-inducible factor (HIF) system and that the complex respiratory responses identified by Haldane and colleagues arise, at least in part, from the interaction of two systems that respond directly to low oxygen: the HIF transcriptional pathway and the arterial chemoreceptors. The arterial chemoreceptors that transduce immediate ventilatory responses to changes in arterial pO_2_, pCO_2_ and other chemical stimuli, were identified more than a decade after Haldane’s work by Corneille Heymans using cross-circulation experiments that isolated the head and trunk of animals except for nerves providing afferent signals to the brain from the aorto-cardiac region. Following detailed studies of its structure and innervation by Fernando de Castro (7, 8), functional studies by Heymans refined the principal site of arterial chemoreceptors, within this region, to the carotid body (9). Several decades later in the 1980s, experiments using isolated perfusion of the carotid body *in situ* in awake goats revealed that the carotid body was also central to the acclimatisation response (10–13). These experiments established that hypoxic, but not hypercapnic, perfusion of the carotid body was necessary and sufficient for an acclimatised response. More accurate characterization of the acclimatisation response in humans was achieved by Peter Robbins and colleagues in Oxford, who used feedback regulation of end-tidal and hence arterial CO_2_ to enable the response to eucapnic hypoxia to be measured (14–16). These studies revealed the acclimatisation response to be much greater under eucapnic than poikilocapnic conditions. Furthermore, as predicted from FitzGerald’s measurements on the extreme sensitivity of the fully acclimatised pCO_2_ to modest change in altitude, in eucapnic conditions ventilatory acclimation could be detected after sustained exposure to even small reductions in inspired oxygen.

Thus, the observations of Haldane’s party at the summit of Pike’s Peak and FitzGerald’s at intermediate altitudes can be resolved by the interaction of two processes, both of which are mediated largely by the carotid body: a rapid response to severe hypoxia (termed the hypoxic ventilatory response, HVR and transduced by oxygen chemoreceptors) and a change in the amplitude of these chemoreceptor responses to both hypoxia and CO_2_. The latter (termed ventilatory acclimatisation to hypoxia, VAH) is activated progressively even in response to very mild hypoxia, and occurs more slowly, on a time-scale of hours or days, compatible with the activation of HIF (5).

**Figure 2:**
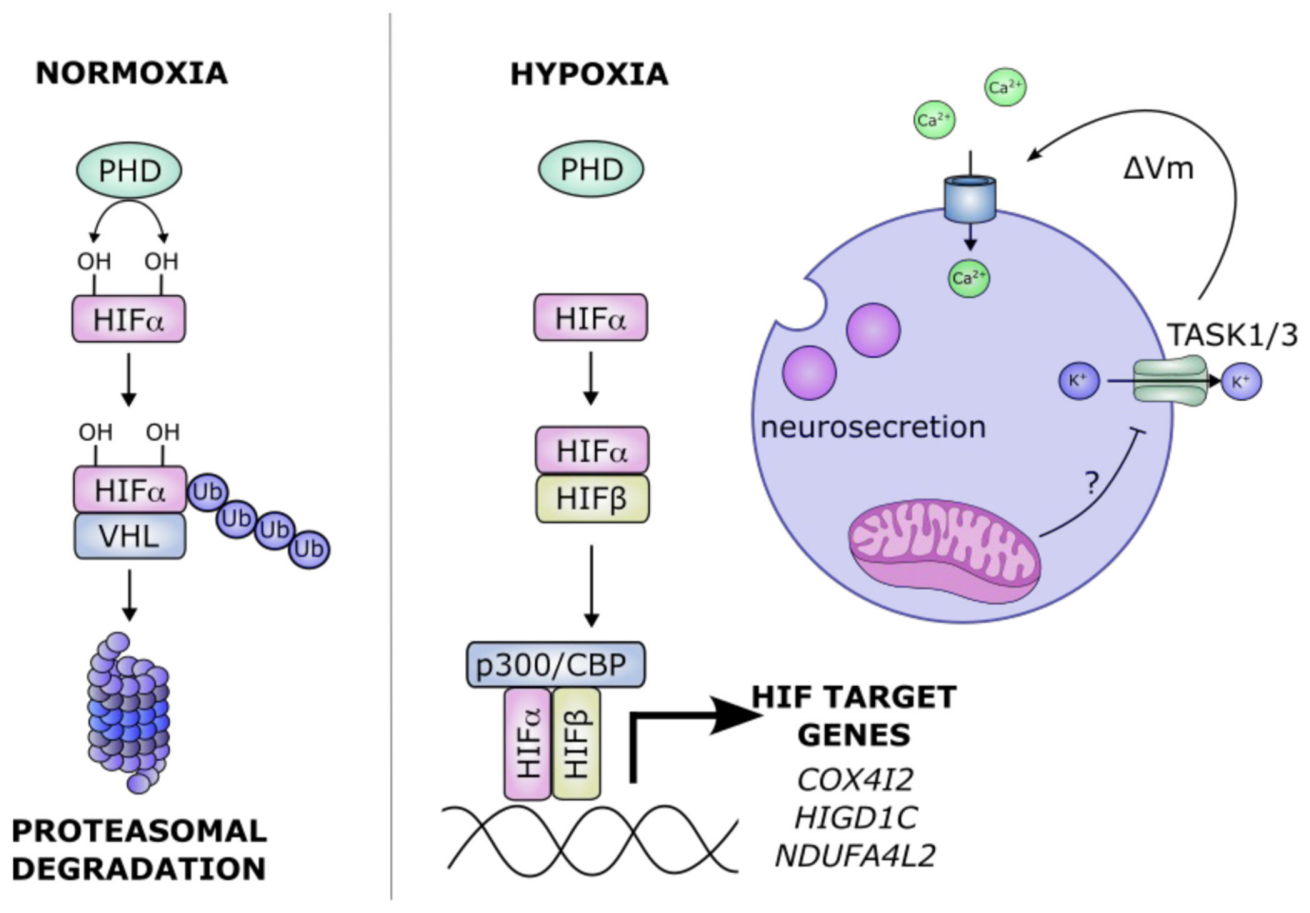
Schematic of the HIF pathway and its potential links with oxygen chemosensitivity. In normoxia, the HIF prolyl hydroxylase enzymes (PHD) hydroxylate HIF-1/2α subunits, allowing for recognition by von-Hippel Lindau (VHL) and targeting for ubiquitin-mediated proteasomal degradation. In hypoxia, HIF-1/2α escapes: PHD-mediated hydroxylation/subsequent degradation and asparaginyl-mediated hydroxylation to allow for the recruitment of transcriptional co-activators p300/CBP (CREB-binding protein), then heterodimerises with HIF**β** to form an active transcription factor that induces the expression of several hundred genes including those potentially involved in oxygen chemosensitivity such as the atypical mitochondrial isoforms: *COX4I2, HIGD1C* and *NDUFA4L2*. These are thought to confer hypoxia sensitivity to mitochondria of carotid body (CB) glomus or Type I cells, resulting in membrane depolarisation (e.g. via TASK1/3 channel inhibition), intracellular calcium influx and dense core vesicle release in response to hypoxia.

Both the mechanism(s) of oxygen chemoreception in the carotid body and of the hypoxia-inducible factor (HIF) transcriptional response have been reviewed elsewhere and the reader is referred to several excellent reviews for the detail of these responses (17–23). In outline, oxygen chemoreceptor function is a cell-restricted property of the Type I cells of the carotid body in which an oxygen-dependent signal, proposed to be generated by the mitochondrion, causes inhibition of K^+^ (likely TWIK-related acid sensitive K^+^, TASK) channels in the plasma membrane (24). This results in membrane depolarization, activation of voltage-gated Ca^2+^ channels and release of transmitters that occurs in response to hypoxia over a time course of seconds (Figure 2). In contrast, hypoxia-inducible factor (HIF) is present in all human and animal cells and transduces a transcriptional cascade of cellular and systemic responses to hypoxia that are activated over a period of hours to days. HIF itself is regulated by post-translational hydroxylation of specific prolyl and asparaginyl residues in its regulatory HIF-α sub-units (21, 25–27). These modifications are catalysed by different members of 2-oxoglutarate dependent dioxygenases superfamily, whose kinetic dependence on oxygen generates the regulatory signals. HIF-α prolyl hydroxylation is catalysed by a set of closely related enzymes termed prolyl hydroxylase domain (PHD) 1, 2 and 3 (encoded by the Egl nine homolog *EGLN 2, 1* and 3 genes respectively), and targets the polypeptide to the von-Hippel-Lindau ubiquitin E3 ligase for proteasomal degradation. HIF-α asparaginyl hydroxylation acts to block CBP/p300 co-activator recruitment and reduce transcriptional activity (25). In hypoxia these processes are suppressed, allowing HIF-α to accumulate, form a DNA binding complex with HIF-β subunits (termed HIF) and activate transcription (Figure 2).

### HIF-2 in ventilatory acclimatisation

The HIF pathway is operative in most, if not all, human and animal cells, but not all components are uniformly expressed. HIF-α, the regulatory sub-unit that specifically transduces responses to hypoxia exists as several isoforms created by gene duplication events at the base of vertebrate evolution (28). In humans, there are three isoforms: HIF-1α, HIF-2α and HIF-3α, of which HIF-1α and HIF-2α are the best studied. Although HIF-1α is widely expressed this is not true of other HIF-α isoforms. In particular, the HIF-2α isoform (originally termed Endothelial Per-Arnt-Sim, EPAS1, protein) is strongly expressed in many of the specialist cells and tissues associated with systemic oxygen delivery in higher vertebrates (29–31). In this review, we will use the term HIF-2α to emphasize the functional role in the biology of hypoxia and relation to the HIF-2α/β dimeric complex.

In the first report of HIF-2α/EPAS1, a ‘gene reporter’ mouse was generated to assess *Hif-2α* transcript expression by replacing the gene with a LacZ reporter; expression was then assessed in a *Hif-2α* heterozygous mouse, since the *Hif-2α*^*-/-*^ genotype is lethal perinatally. This analysis demonstrated marked cell type specific expression, with high levels along the developing sympathetic chain and strikingly high expression in the carotid body of adult mice (29). This distinct expression profile was subsequently corroborated almost twenty years later by transcriptomic studies which showed that *Hif-2α* mRNA is one of the most abundant transcripts in the carotid body and in particular in the Type I chemosensitive cells (as assessed by single cell transcriptomics), as well as one of the most differentially expressed when compared against related sympathoadrenal cells such as neuronal cells of the superior cervical ganglia (32, 33).

**Figure 3:**
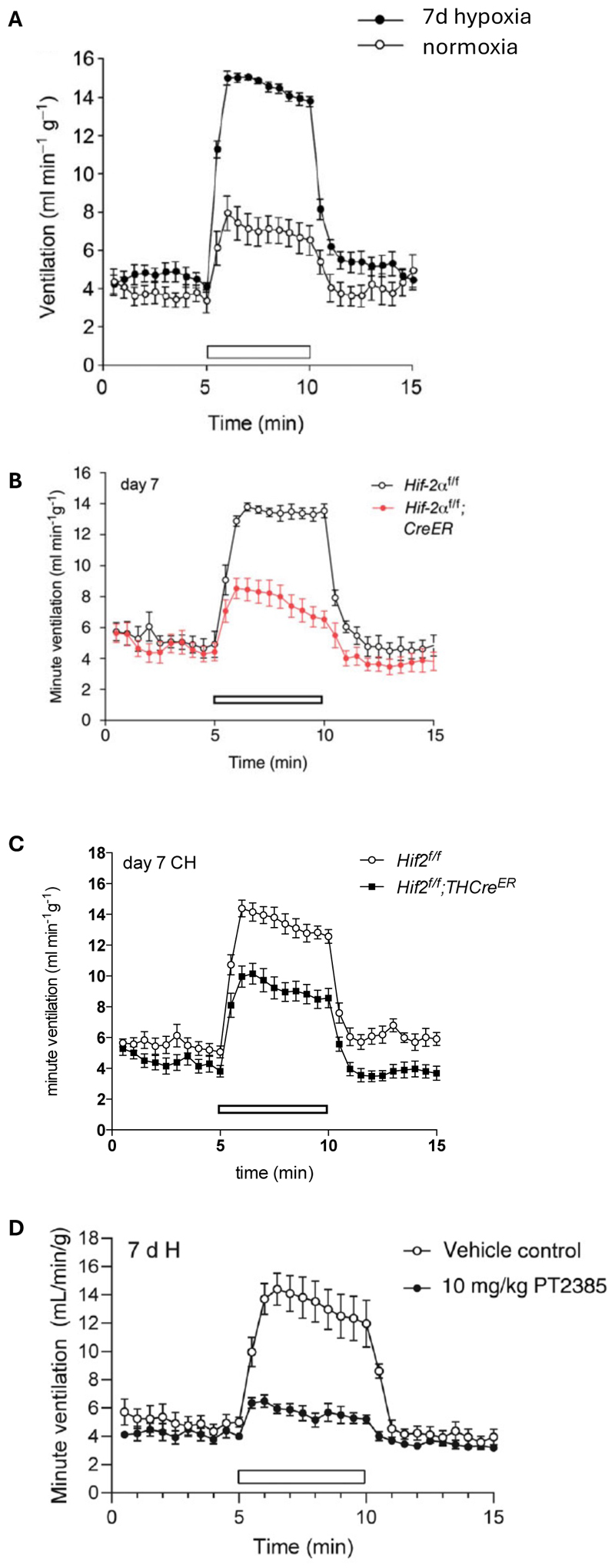
HIF-2α is necessary for ventilatory acclimatisation. **(A)** Enhanced ventilatory responses to acute hypoxia (white bars) in mice exposed to 7 days at 10% oxygen (termed ventilatory acclimatisation). Inactivation of HIF-2**α** in adult mice, either ubiquitously across tissues in tamoxifen treated *RosaCreER* mice **(B)** or restricted to tyrosine hydroxylase (TH) specific tissues in tamoxifen treated *THCreER* mice **(C)**, ablates the ventilatory acclimatisation observed in (A). Pharmacological antagonism of HIF-2**α** using PT2385 ablates ventilatory acclimatisation, mimicking the effects of genetic inactivation noted in (B) and (C). Data from Bishop *et al*., *JPhysiol*, 2013; Hodson *et al*., *JPhysiol*, 2016; Fielding *et al*., *JPhysiol*, 2018; Cheng *et al*., *JCI*, 2020.

Evidence for heightened ventilatory sensitivity to hypoxia associated with general activation of HIF was first obtained in humans (and later in mice) with Chuvash Polycythaemia (bearing a hypomorphic mutation in the von Hippel-Lindau gene, *VHL*) and then in mice that were heterozygous for inactivation of the principal HIF prolyl hydroxylase (*Phd2*)(34–36). Following this, a functional role for HIF-2α in hypoxic ventilatory control was demonstrated using transgenic mice, where loss of *Hif-2α* (that is restricted to adult mice to avoid any confounding developmental effects), either ubiquitously across cell types, or specifically in tyrosine hydroxylase positive (TH^+^) cells, ablates ventilatory acclimatisation as well as carotid body Type I cell proliferation (Figure 3)(37, 38). In contrast, loss of *Hif-1α* in these contexts had no effect. HIF-2α, but not HIF-1α, was also found to mediate increased hypoxia ventilatory responses achieved by inactivation of *Phd2* (rather than chronic hypoxia) (38). This work is apparently in conflict with earlier findings where *Hif-1α* heterozygous mice were found to manifest a small reduction in ventilatory acclimatisation (39). The reasons for the discrepancies between these studies are not clear.

Confirmation for the role of HIF-2α in the carotid body was obtained pharmacologically using HIF-2α specific antagonists. Transcription factors are typically very challenging to target pharmacologically, but this was achieved for HIF-2α by exploiting features of its Per-Arnt-Sim (PAS) B domain, which has a significantly larger cavity than that of HIF-1α thus allowing specific targeting of the HIF-2α isoform. This class of drugs bind to the PAS B domain to reduce heterodimerisation with HIF-β (also known as aryl hydrocarbon receptor nuclear translocator, ARNT)(40, 41). HIF-2α antagonist treatment of adult mice was found to almost completely ablate ventilatory acclimatisation and carotid body proliferation (Figure 3)(42). Thus, an orthogonal approach using a pharmacological, rather than genetic, intervention demonstrates that HIF-2 mediates hypoxia responses in the carotid body.

Interestingly, parallel studies in transgenic mice have shown that HIF-2α, not HIF-1α, is also the important isoform in mediating chronic hypoxia (and *Phd2ko*) induced polycythaemia (38, 43). *Hif-2α* is highly abundant in renal interstitial fibroblasts which produce erythropoietin to mediate this erythropoiesis (44, 45), mirroring the abundant expression of *Hif-2α* seen in the carotid body Type I cells which mediate hypoxic ventilatory control (Figure 1B, C)(37). Thus, the vertebrate-specific HIF-2α paralogue, whose appearance coincides with the evolution of complex oxygen delivery systems, plays a crucial role in regulating two key components of those systems: oxygen loading of the blood by the lungs and oxygen carriage by red blood cells.

### HIF-2 in oxygen chemosensitivity

The role of HIF-2α in ventilatory acclimatisation, a response largely mediated by the peripheral chemoreceptors in the carotid body, raised a further question as to the role of HIF-2α in chemoreceptor function itself.

Indeed, it was observed that inactivation of *Hif-2α* not only ablates ventilatory acclimatisation but also dampens hypoxic ventilatory control in unacclimatised mice, suggesting that HIF-2α acts at the interface between transcriptional responses to hypoxia and classical chemosensitivity (37, 38, 42). Work from Jose Lopez-Barneo and colleagues confirmed this finding with ubiquitous *Hif-2α* inactivation in adult mice and further showed that loss of *Hif-2α* ablates electrophysiological oxygen chemosensitivity of Type I cells (46). In addition to these findings on inducible inactivation of *Hif-2α* in adult life, constitutive inactivation of *Hif-2α* in TH^+^ cells, results in arrest of carotid body development and a vestigial organ (37, 47).

One possibility is that HIF-2α is involved directly or indirectly in the transcription of critical components of the oxygen chemosensory apparatus (Figure 2). Type I carotid body cells have specialised mitochondria, which have reduced oxygen affinity compared to related neuronal cells of the superior cervical ganglion (SCG) (48–50). This unusual property has been attributed to the high expression of the atypical mitochondrial subunits: *Cox4i2, Higd1c* and *Ndufa4l2* in Type I cells relative to neuronal cells of the SCG and other cell types (32). Expression of these atypical mitochondrial subunits, which are all electron transport chain complex IV-interacting proteins, may therefore be the distinguishing feature of Type I cells that enhances complex IV sensitivity to hypoxia. In line with this, co-expression of HIGD1C together with COX4I2 reduces oxygen affinity in cells (two-fold increase in p50 for oxygen)(51). HIF-2 was found to regulate the expression of *Cox4i2* and *Ndufa4l2* and inactivation of *Cox4i2* or *Higd1c* (but not *Ndufa4l2*) phenocopies that of *Hif-2α* in ablating oxygen chemosensitivity at the cellular level (calcium responses to hypoxia) and at the whole animal level (plethysmographic measurement of respiration) (46, 51). This suggests that both COX4I2 and HIGD1C are necessary for oxygen chemosensitivity and that the role for HIF-2 in this process is via the transcriptional induction of *Cox4i2* and *Higd1c* in the carotid body (23)(Figure 2). However, some puzzling features remain unexplained - raising the possibility that not all actions of HIF-2α on the carotid body are transcriptional.

When interventions are made on *Hif-2α*, changes in physiology such as HVR do not always correlate well with changes in *Cox4i2* transcript levels, possibly reflecting a developmental (as opposed to adaptive) role for HIF-2-mediated transcription. However, it is also possible that HIF-2α has additional non-transcriptional role(s) in oxygen chemosensing. A transgenic mouse carrying a point mutation (S305M) in the PAS B domain that blocks HIF-2α antagonist binding without interfering with its ability to heterodimerise with ARNT nevertheless had reduced hypoxic ventilatory responses (42), as well as reduced hypoxia-induced calcium entry in isolated CBs (unpublished observations). A similar hypofunctional phenotype for this point mutant *Hif-2α* with respect to erythropoiesis has also been reported (52). Thus, it is possible that modulating the PAS B domain impairs HIF-2 function, independently of its ability to heterodimerise with ARNT and activate targest gene expression. In keeping with this, *Hif-2α* mRNA levels are, unexpectedly, very high in the CB, in marked excess compared to other transcription factors and its dimerization partners: ARNT1 or ARNT2 (32, 33), suggesting that HIF-2α may also act independently of dimerization and DNA binding.

Together, these findings suggest that HIF-2α may have a role in oxygen chemosensitivity beyond as a canonical transcription factor. The presence of a large PAS B domain that is specific to HIF-2α raises the interesting possibility that endogenous ligand/s may exist to modulate HIF-2α function, and potentially in a non-transcriptional manner. Indeed, endogenous, small molecule ligands have been reported which bind to the PAS domain of related proteins. For example, the lipid oleoylethanolamide binds the PAS B domain of HIF-3α to modulate activity (53). A further example is the aryl hydrocarbon receptor (AHR), a protein in the same transcription factor family as HIF-2α, which is activated by binding of a range of endogenous ligands to its PAS B domain (e.g. arachidonic acid, pyrene, tryptophan and flavonoid derivates such as the neuroendocrine modulators serotonin and melatonin)(54). It is therefore possible that HIF-2α may also have an endogenous ligand whose regulation contributes to oxygen sensitivity.

### Evolutionary adaptation to high altitude: titrating the HIF-2 response

Many of the physiological responses that enable acclimatisation to hypoxia can become pathological if hypoxia is sustained, for example, with prolonged residence at high altitude. These may manifest as chronic mountain sickness (CMS) characterised by excessive erythropoiesis and pulmonary hypertension (55). The latter results from sustained hypoxia-induced pulmonary vasoconstriction and vascular remodelling.

Hypoxia induces time-limited, HIF-2 dependent proliferation of several cell types within the lung: both epithelial and endothelial, though the functional relevance of this response is not clear (56). HIF-2α is abundantly expressed in the lung in epithelial as well as endothelial cells (44) and mice with genetic inactivation of *Hif-2α* in pulmonary endothelial cells are protected from pulmonary hypertension induced by chronic hypoxia or genetic inactivation of *Phd2* (57, 58). Thus, sustained HIF-2 activation in hypoxia appears to mediate maladaptive (pulmonary hypertension and excessive erythropoiesis), as well as adaptive (ventilatory acclimatisation and erythropoiesis), responses to altitude.

Several human populations (Tibetans, Andeans, Ethiopians), and many other mammalian sub-species, have lived at high altitude for thousands of years and have adapted to that environment, being relatively protected from CMS (reviewed in (59)). The HIF pathway is a natural focus for evolutionary or genetic strategies for hypoxia adaptation as it is a central molecular hub co-ordinating animal oxygen homeostasis. However, this critical role may constrain evolutionary variation and it is notable that the core HIF pathway is highly conserved across the animal kingdom and that there are few, if any, high-altitude variants of *HIF-1α*. Gene duplication in the HIF pathway and the appearance of the *HIF-2α* isoform during vertebrate evolution (28) provides more freedom to regulate specific processes in systemic physiology. Indeed, GWAS of altitude populations has revealed strong selection signals at the *EPAS1* locus across species and geographic regions (60).

High altitude variants occur in different locations in *EPAS1*, both within the coding region: Tibetan horses (R144C and E263D)), human Andeans (H194R), Tibetan dogs (G305S)(61), North American deer mice (T755M), as well as in the non-coding region for human Tibetans (62, 63). What is the directionality of these SNP effects: hypo (or hyper)-responsive? Are all the physiological responses dampened across all HA populations or is protection against different processes selected in different populations?

Accurate molecular and physiological phenotyping is needed to answer these questions. For example, the human Andean H194R variant in the PAS A domain impairs heterodimerisation with ARNT, leading to decreased transcription and reduction of right ventricular hypertension in a mouse model, suggesting that it is a hypofunctional allele (64). The deer mouse T755M variant impairs binding of the transcriptional coactivator CREB-binding protein (CBP) to reduce transcription and impairs both VAH and CB hyperplasia (without affecting erythropoiesis) in a mouse model, again suggesting a hypofunctional allele (65, 66). Allelic variation in the non-coding region of *EPAS1* in human Tibetans is associated with lower haemoglobin and erythropoietin levels (63), suggesting reduced HIF-2α function, by reduced expression of *HIF-2α*. Of note, the high-altitude variant in Tibetan dogs resides in the PAS B domain (G305S)(61), supporting the notion discussed earlier that this domain has an important functional role, perhaps through binding of an endogenous ligand.

Thus, reduced HIF-2α activity is a common adaptive response in high altitude populations. However, the location of the variants, molecular mechanisms and physiological processes affected vary, suggesting the operation of a form of convergent evolution across different high altitude adapted populations.

Evolution must operate at the level of reproductive fitness. This may be impacted by adult survival, reproductive behaviours or embryonic viability. It is well established that hypoxia has marked effects on embryonic viability. For instance, unphysiological HIF activation results in death from placental and cardiac defects in *Phd2*^*-/-*^ mice (67). Effects of hypoxia on pregnancy outcome are apparently mitigated in high altitude adapted populations who have normal reproductive capabilities (in contrast with the Spanish settlers living at altitude in Peru who often failed to reproduce successfully, known as the Conquistadors’ curse (68)). An interesting question is whether reproductive adaptation could also be HIF-2-dependent. Challenges to oxygen homeostasis are also a feature of the perinatal period, when the fetus is subject to intermittent hypoxia (e.g. from uterine contractions) and then must transition from placental to pulmonary gas exchange and air breathing life. Normal carotid body physiology is critical to survival during this period, being necessary for fetal hypoxia tolerance (69) and establishment of stable ventilatory control. Again, through its role in CB function, perturbations in HIF-2 activity might impact perinatal survival.

In summary, there is substantial evidence for a selection pressure to modify HIF-2 activity as an adaptation to environmental hypoxia. The modifications vary both genetically and in their physiological activity in line with the broad reach of physiological control by this pathway.

### Hif-2 and the inheritance of hypoxia pathway tumours

Thus, the vertebrate-specific HIF-2α isoform is central to the maintenance of several of the key vertebrate oxygen uptake and delivery systems. But perhaps the most striking clinical manifestation of *HIF-2α* mutations are in neuroendocrine tumours of autonomic paraganglia known as paragangliomas (PGLs).

PGLs are located from the base of the skull to the pelvis, including in the carotid body and adrenal medulla (where they are termed pheochromocytoma). Although they are genetically diverse, with over ∼20 ‘driver’ genes identified to date, in a substantial proportion the mutations directly or indirectly affect hypoxia signalling (70, 71). These include gain of function mutations in *HIF-2α* itself as well as loss of function mutations in negative regulators of HIF: *VHL, PHD2* and *PHD1*. The most commonly observed mutations affect the tricarboxylic acid cycle enzyme, succinate dehydrogenase (*SDHB*/*D*/*C*/*A* or *SDHx*), in particular *SDHB* (72). These metabolic mutations lead to the accumulation of succinate, which inhibits 2-oxoglutarate dependent dioxygenases including the PHD enzymes and the ten-eleven translocation (TET) enzymes, which normally promote demethylation of DNA via oxidations of 5-methylcytosine bases. HIF-2α activation, together with TET inhibition, has been shown to recapitulate the *SDHB* metastatic phenotype (73). Thus, mutations in all these genes (*SDH, HIF-2α, VHL, PHD2, PHD1*) result in unphysiological activation of HIF-2α, or a ‘pseudohypoxic’ phenotype in the absence of hypoxia *per se*. Other connections to hypoxia signalling have been identified in epidemiological studies. Hypoxia (high altitude or chronic hypoxaemia due to cyanotic congenital heart disease) also increases the risk of developing PGL tumours, most strikingly carotid body paragangliomas (74–80). These tumours are up to 10x more frequent in high altitude populations. Interestingly, gain of function mutations affecting *HIF-2α* may also interact with hypoxia, as these mutations have been reported in 80% of patients with PGL in the context of cyanotic heart disease (74), in contrast to 5-6% of all PGL tumours. Taken together, these findings provide very strong evidence for the causality of HIF activation in this unusual type of oncogenesis. Paradoxically, despite strong association between malignancy and microenvionmental or indirect activation of HIF across multiple types of cancer, directly activating HIF mutations are not seen in cancer other than PGL (and rarely in somatostatinomas, a neuroendocrine tumour which can present alongside PGLs (81)). A further curious feature of PGLs is that they are very often heritable (up to 40% are associated with a germline mutation)(82).

**Figure 4:**
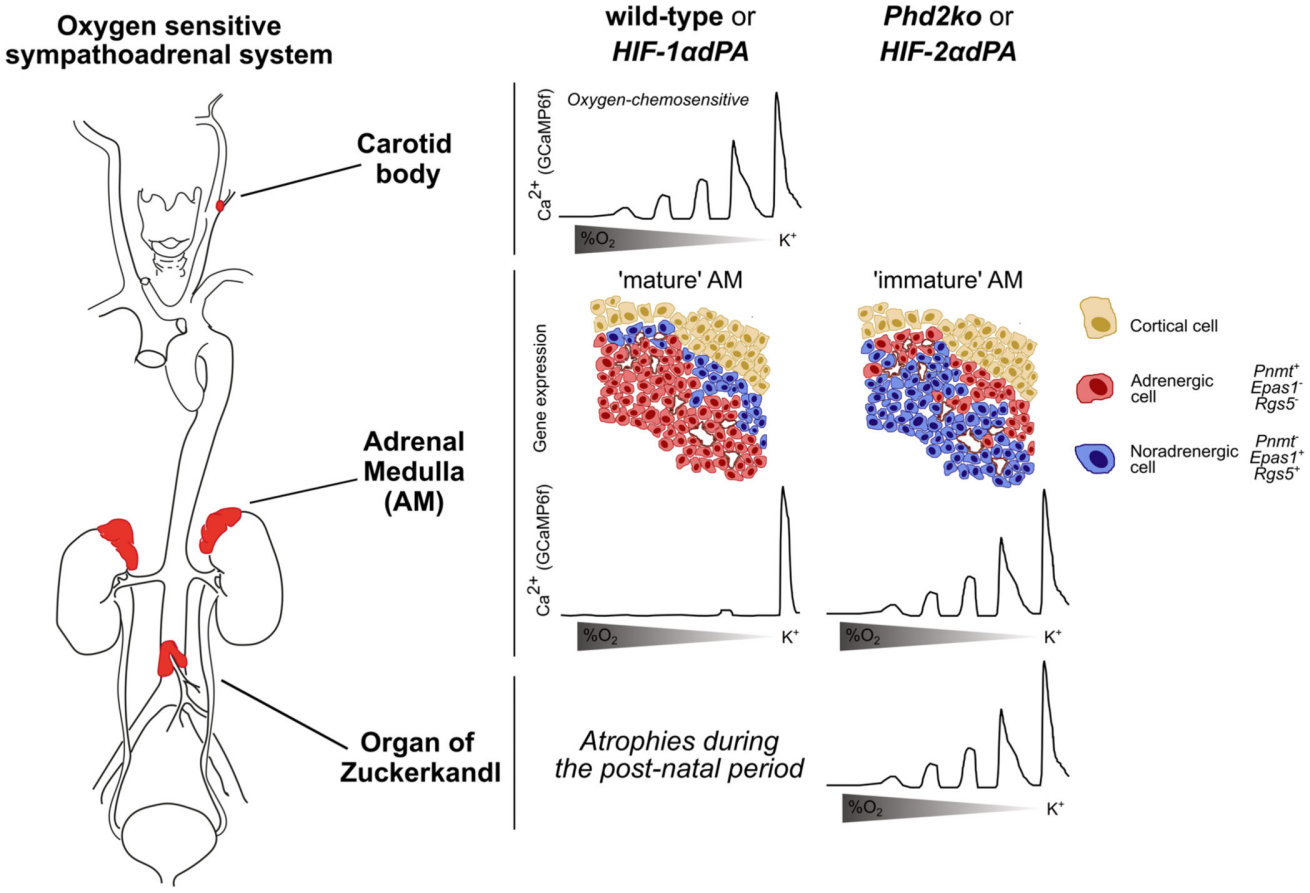
Developmental *Phd2* inactivation or *Hif-2α* activation results in the retention of fetal chroma7in cells that are oxygen chemosensitive. Oxygen chemosensitivity is observed in adult chroma6in cells of the carotid body, but not at other sites including the adrenal medulla, which is principally adrenergic (*Pnmt*^*+*^) and lacks *Epas1*/*Hif-2**α*** and *Rgs5* mRNA as well as the Organ of Zuckerkandl (which regresses post-natally and is absent in the adult). *Phd2* inactivation that is restricted to chroma6in cells by tyrosine hydroxylase (TH) promoter driven Cre recombinase (*Phd2*^*f/f*^*;ThCre* or *Phd2ko*) results in: the retention of the fetal Organ of Zuckerkandl; a population switch towards adrenergic (*Pnmt*^*-*^) chroma6in cells in the adrenal medulla which express the carotid body enriched genes *Epas1* and *Rgs5*. Further, chroma6in cells from both *Phd2ko:* adult adrenal medulla and Organ of Zuckerkandl are oxygen chemosensitive, similar to the carotid body. These morphological and oxygen chemosensitive phenotypes can be recapitulated by the expression of stabilised *Hif-2* (but not *Hif-1*)***α***. This was achieved by replacing the two hydroxylated prolines in *Hif**α*** with alanine residues, expressed at the *Rosa26* locus and preceded by a restricting ‘*lox-stop-lox*’ sequence (Kim *et al*., *EMBO J*, 2006). These mice were intercrossed with mice expressing Cre recombinase under a tyrosine hydroxylase (TH) promoter to generate either *Hif-1 or 2* *****α******dPA* mice. Graphical abstract from Prange-Barczynska *et al*., *JCI*, 2024.

Some light on these findings is potentially provided by experimental studies of the genetic manipulation of the HIF pathway in mice. We have modelled pseudohypoxic PGL by inactivation of *Phd2* (and hence activation of HIF) in TH^+^ sympathoadrenal cells (37, 83, 84). This results in PGL-like morphology with greatly enlarged, dysmorphic CBs. Most interestingly, however, *Phd2* inactivation in TH^+^ cells also led to retention of immature, fetal-like chromaffin cells: persistence of the fetal organ of Zuckerkandl (OZ) and of immature chromaffin cell populations both within and adjacent to the adrenal medulla (Figure 4). These cells do not express PNMT, the final enzyme in the catecholamine synthesis pathways that synthetizes adrenaline from noradrenaline, hence they secrete noradrenaline. The changes could not be mimicked by inactivation of *Phd2* in the adult, suggesting that inactivation of *Phd2* sometime during development is critical. Alterations were reversed by combined inactivation of *Hif-2α* but not *Hif-1α* (37, 83). Furthermore, overexpression of a stabilized form of HIF-2α, but not HIF-1α, was found to be sufficient to generate the phenotype (84)(Figure 4). Taken together with failure of development of the carotid body following *Hif-2α* inactivation in TH^+^ cells (37, 47), these findings suggest that the oncogenic effects of HIF pathway activation in pseudo-hypoxic PGL may arise from a developmental action of HIF-2α on the sympathoadrenal system. In contrast with the general role of the HIF pathway in adaptive responses to hypoxia, such an activity on differentiation might be predicted to be intrinsically tissue restricted. Potentially this would explain both the high prevalence of heritable germline mutation and the tight tissue restriction of pseudo-hypoxic PGL.

What mechanisms might contribute to HIF-2 driven oncogenesis? Transcript profiling by RNA-seq revealed that many of the genes that are upregulated by *Phd2* inactivation (and hence HIF activation) in the adrenal medulla are also enriched in the carotid body (84). These include HIF-2α itself, molecules with roles in G-protein signalling pathways (e.g. the regulatory protein RGS5 and the receptor ADORA2A) and the atypical regulatory subunits of cytochrome *c* oxidase that have been implicated in oxygen chemosensitivity itself (see above). Furthermore, single cell transcriptomic studies of developing chromaffin cells have also identified an ‘oxygen sensing cluster’ of cells expressing *Hif-2α*, as well as the alternative cytochrome oxidase subunits *Cox4i2* and *Ndufa4l2* (85, 86). Most strikingly, however, using genetically encoded calcium indicators to measure rapid electrophysiological responses to hypoxia, it was observed that both inactivation of *Phd2* and overexpression of HIF-2α confer functional oxygen chemosensitivity in these immature chromaffin cells similar to that reported in the fetal adrenal gland, and akin to that in the carotid body (84)(Figure 4).

Might this induction of chemosensitivity underlie the predisposition to tumourigenesis? There are interesting parallels between the normal physiology of the carotid body response to hypoxia and the pathological consequences of pseudohypoxia exhibited by PGL tumours. The carotid body has the unusual property of initiating a striking cellular proliferative response to sustained hypoxia, resulting in organ hyperplasia upon chronic hypoxia exposure. Hypoxia-induced proliferation appears to be connected in an as yet unknown way to ventilatory acclimatisation, as interventions that impair VAH also ablate the CB proliferative response and both are governed by the PHD2/HIF-2 axis (38). This raises the interesting question as to whether chemosensitivity and connections to the associated proliferative response might, if dysregulated, drive oncogenesis. In line with this, it has previously been proposed that the neurosecretory activity of sympathoadrenal cells may promote their growth (87). Whatever the mechanism of oncogenesis, the findings on the ability of HIF-2α to promote chemosensitivity in sympathoadrenal cells have raised other interesting biological and medical questions.

First, does the retention of chemosensitivity contribute to the symptomatology of PGLs i.e. does hypoxia stimulate noradrenaline secretion and potentially trigger hypertensive crises? There is a case report of a male with a diagnosed noradrenergic PGL (without *SDHB* or *D* mutations but otherwise genetically undiagnosed) experiencing a hypertensive crisis with very elevated normetanephrines triggered by an ascent of Mount Kilimanjaro (88). If the connection were causal, this would suggest that it could be important to monitor and avoid hypoxaemia in such patients.

Second, is HIF-2α induced oxygen chemosensitivity a generalised property across pseudo-hypoxic PGLs? Current mouse models bearing these other pseudo-hypoxic mutations have not generated PGL (89) and CBs and AMs are absent in when *Vhl* is inactivated in TH^+^ cells (90). This may suggest that more precise quantitative actions on HIF pathway activation are important. In humans, Type I (null) *VHL* mutations, which the mouse *Vhlko* likely models, do not develop PGLs; these are only observed with Type IIB/C *VHL* point mutations, which manifest strikingly less upregulation of the HIF pathway (91). In the case of SDHx, various mouse models have also been created for each of *Sdhb*/*d*/*c*, but none have resulted in PGLs. Again, this may be because inactivation does not mimic disease-associated human mutation precisely. Evidence that precise dysregulation of hypoxia signalling is required for PGL oncogenesis is provided by the *HIF-2α* mutational spectrum associated with PGL which centres on a ‘hot-spot’ in the vicinity of the hydroxylated proline 531 residue (∼80% of *EPAS1* mutations in PGLs lie between αα529-532). In contrast, mutations resulting in polycythaemia are also located at a hotspot, but oddly, at a nearby but different site αα533-549 (92). Together this suggests that the phenotypic response is finely tuned to the location of the mutation, perhaps because differences in the latter result in different levels of HIF-2α activation.

In the meantime, the first report of treatment of a patient with a *HIF-2α* gain-of-function PGL with the HIF-2α antagonist Belzutifan recorded a very rapid reduction in plasma chromogranin A and normetanephrines within 9 days of starting treatment, followed by a reduction in tumour size within 17 days that was sustained for 24 months (93). This suggests that HIF-2α does indeed regulate secretion from PGLs together with anti-tumour activity and that further analyses of the more general role of HIF-2α in pseudo-hypoxic PGL is urgently needed.

## Conclusions

The work that we have reviewed defines a non-redundant role for HIF-2α in the physiology and pathophysiology of the carotid body and related sympathoadrenal tissues. HIF-2α is essential for the development and maintenance of chemoreceptor function in the carotid body. It is required for the enhancement of chemosensitivity that characterizes ventilatory acclimatisation to hypoxia and provides a molecular explanation for many of the initially puzzling findings of progressive increases in ventilation at altitude that were observed by early investigators. HIF-2α also appears to be capable of driving the development of paraganglioma possibly due to actions on the developing sympathoadrenal system. Further work is required to define the precise mechanisms connecting these actions. However, in keeping with a very precise regulatory action of HIF-2α, both developmental and oncogenic actions appear to require precisely tuned levels of activation. This is also reflected in the action of human and animal polymorphisms at the *HIF-2α* locus which appear to moderate aspects of the hypoxia response and to have been selected in altitude adapted populations.
